# A Comparative Analysis of Quadriceps Tendon, Patellar Tendon Bone Allograft, and Cadaver Graft in Anterior Cruciate Ligament (ACL) Repair and Reconstructive Surgery

**DOI:** 10.7759/cureus.59836

**Published:** 2024-05-07

**Authors:** Brandon Krumbach, Christopher Meretsky, Anthony T Schiuma, Mohammed Ajebli

**Affiliations:** 1 Anatomy, St. George's University, Great River, USA; 2 Medicine, St. George's University Medical School, True Blue, GRD; 3 Orthopedic Surgery, Holy Cross Hospital, Fort Lauderdale, USA; 4 Biology Sciences, Faculty of Sciences and Technology, Moulay Ismail University, Errachidia, MAR

**Keywords:** sport's knee injury, injury, anterior cruciate ligament (acl) injuries, acl injury, knee ligamentous reconstruction, ligament reconstruction, athlete's knee, acl tear, anterior cruciate ligament (acl) reconstruction

## Abstract

Anterior cruciate ligament (ACL) injuries are a common occurrence among athletes and active individuals, often necessitating surgical intervention for optimal recovery. The choice of graft material for ACL reconstruction remains a topic of debate, with various options available, including quadriceps tendon (QT), patellar tendon bone allograft (PTBA), and cadaver graft (CG). This paper aims to provide an extensive review and comparison of the efficacy, outcomes, and complications associated with these graft types based on recent research. A systematic literature search following PRISMA guidelines was conducted to identify relevant studies published in the past six years. The findings suggest that while each graft type has its advantages and limitations, there is no definitive superior choice. Factors such as patient age, activity level, comorbidities, and surgeon preference should be considered when selecting the most appropriate graft for ACL repair surgery. QT grafts are associated with lower donor-site morbidity compared to patellar tendon grafts. However, QT grafts may have a higher risk of graft rupture and decreased knee flexion strength. PTBA grafts, compared to QT grafts, have a higher risk of donor-site morbidity but a lower risk of graft rupture and improved knee stability. CG grafts have lower donor-site morbidity compared to PTBA grafts but may have a higher risk of graft rupture and decreased knee flexion strength compared to PTBA grafts. In conclusion, the choice of graft material for ACL reconstruction is a complex decision that requires careful consideration of various factors, including patient age, activity level, comorbidities, and surgeon preference. While each graft type has its advantages and limitations, there is no definitive superior choice. Therefore, it is essential to carefully weigh the risks and benefits of each graft type to ensure optimal outcomes for patients undergoing ACL repair surgery.

## Introduction and background

Anterior cruciate ligament (ACL) repair surgery is a critical procedure aimed at restoring knee stability and function following ACL injuries [1]. One of the most crucial decisions surgeons face during ACL reconstruction is the selection of the ideal graft type [2]. Three main options exist: allografts, patellar tendon autografts, and hamstring tendon autografts (often referred to as cadaver grafts (CGs), which can encompass both sources). Each graft type presents unique advantages and disadvantages that surgeons must carefully weigh to optimize surgical outcomes and patient recovery [3]. Allografts, sourced from human cadaveric donors, offer a convenient alternative to autografts. They eliminate the need for a second surgical site to harvest a tendon, minimizing donor site morbidity and potentially leading to shorter operative times [4,5]. However, allografts may have a higher risk of disease transmission, slower incorporation into the recipient's tissues, and potentially higher rates of graft failure, particularly in younger and more active patients [6]. This slower incorporation can lead to a longer period of knee instability and delayed return to full activity. Patellar tendon autografts, harvested from the patient's own kneecap, boast a long history of success, particularly in high-demand scenarios [7]. Their inherent strength and reliable fixation techniques contribute to lower failure rates compared to some allografts [8]. This makes them a popular choice for professional athletes and young, active individuals seeking a rapid return to pre-injury function. However, patellar tendon autograft surgery can lead to donor site morbidity, including pain, weakness, and kneeling discomfort, particularly in the long term [9]. CGs, also known as allografts, are harvested from the tendons of deceased donors and offer an alternative to autografts. They are commonly used in several scenarios: revision ACL reconstruction (re-surgery after a previous ACL repair), multi-ligament knee injuries involving damage to multiple stabilizing structures, and for individuals with lower activity levels who may not require the robust strength of an autograft [2]. They eliminate the need for a separate surgical site to harvest a tendon, avoiding donor site morbidity associated with autografts [10]. Additionally, allograft availability is not limited by the patient's own anatomy, ensuring a readily available tissue source for surgery [11]. However, some drawbacks are associated with allografts. The risk of disease transmission, although rigorously minimized through donor screening and tissue processing, is a concern compared to autografts [12]. Furthermore, allografts may integrate into the recipient's tissues at a slower pace compared to autografts, potentially leading to a longer period of instability and delayed return to function, particularly in younger, highly active patients whose bodies demand a faster healing response [13]. While, the quadriceps tendon (QT) autograft technique for ACL reconstruction has evolved significantly. Earlier procedures involved harvesting a bone block from the kneecap (patella). In 1998, Fulkerson introduced a refined technique using a free QT graft without the bone block [14].

Generally, each graft type has its advantages and disadvantages in terms of surgical technique, graft incorporation, recovery time, and risk of complications. Understanding the comparative efficacy and outcomes of different graft materials is crucial for informed decision-making in ACL reconstruction surgery. Obviously, the choice of graft in ACL repair surgery is a crucial decision that should be individualized based on patient-specific factors, surgical considerations, and desired outcomes. Understanding the nuances of allografts, patellar tendon autografts, CGs, and QT autograft is essential for orthopedic surgeons to tailor the surgical approach to each patient's unique needs and optimize the long-term success of ACL repair procedures. The aim of this review is to perform a comparative analysis of QT, patellar tendon allograft, and CG in ACL reconstruction and repair surgery based on recent studies including case reports, randomized controlled trials, cohort studies, case series, and technical notes.

## Review

Materials and methods 

**Figure 1:**
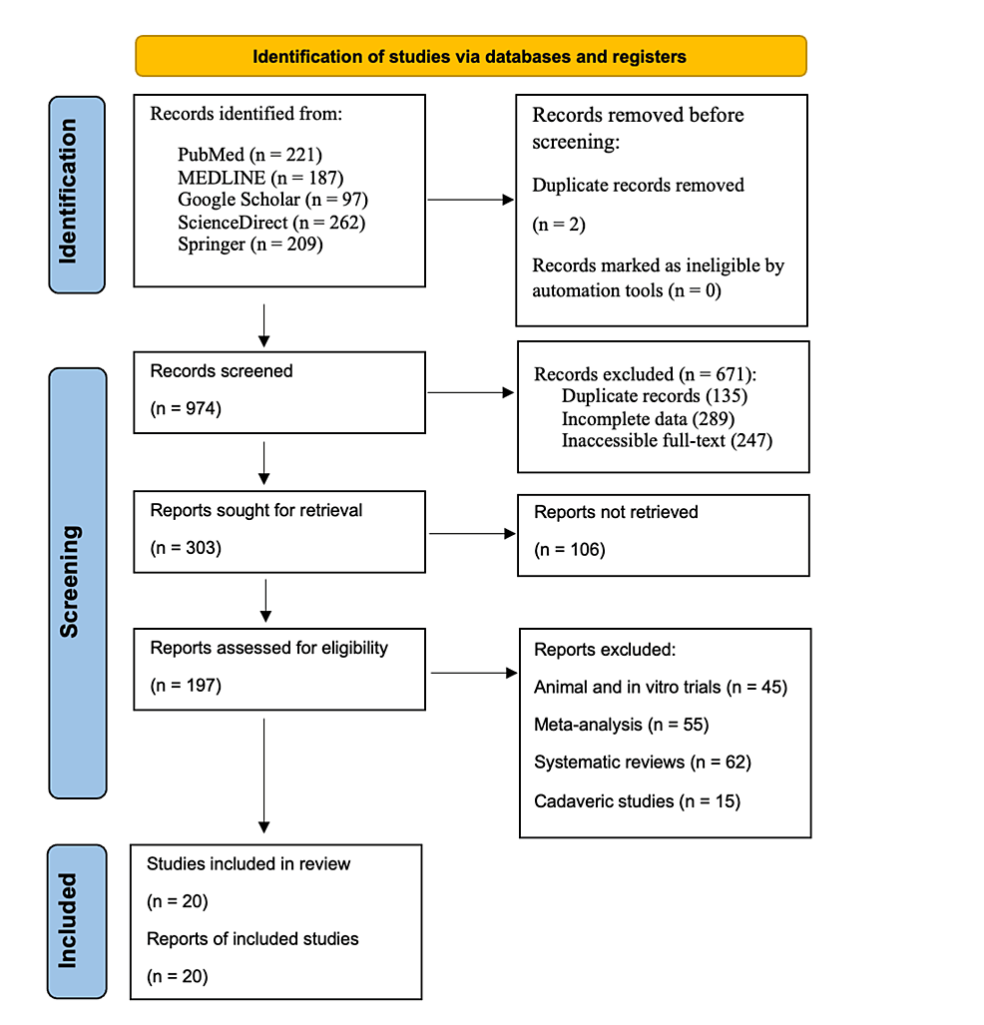
PRISMA flow chart (literature search and study selection). Following the PRISMA guidelines, our search was conducted using the academic databases PubMed, MEDLINE, Google Scholar, ScienceDirect, and Springer with relevant keywords focused on the efficacy of different materials used in ACL graft repairs. We included studies published over a 6-year period (2018-2024) that met the inclusion criteria for this review paper. n: number; PRISMA: Preferred Reporting Items for Systematic Reviews and Meta-Analyses; ACL: Anterior cruciate ligament.

A systematic literature review was conducted using electronic databases, including PubMed, MEDLINE, Google Scholar, ScienceDirect, and Springer, to identify relevant studies comparing QT, patellar tendon bone allograft (PTBA), and CG in ACL reconstruction and repair surgery. The search strategy utilized a combination of keywords related to ACL reconstruction, graft types, outcomes, and complications. Studies published recently (2018-2024) were included to capture recent advancements and trends in ACL surgery. The search was limited to English-language articles involving human subjects. Figure 1 describes the search approach and studies selected for this systematic review using the Preferred Reporting Items for Systematic Reviews and Meta-Analyses (PRISMA) 2020 flow diagram [15].

After the data was collected and evaluated, the research team determined the study objectives, analysis methods, and inclusion and exclusion criteria. This approach is not recommended as it can introduce bias and limit the validity of the study. Ideally, these elements should be established before data collection to ensure a rigorous and unbiased research design. By determining the objectives, analysis methods, and inclusion and exclusion criteria after data collection, the study may be compromised and the results may not be generalizable.

Study selection criteria and process 

Inclusion Criteria

The inclusion criteria for this study encompass randomized controlled trials, case reports, case studies, cohort studies, and technical notes that compare QT, PTBA, and CG in ACL reconstruction and repair surgeries. Additionally, the study includes research that focuses on adult patients who have undergone ACL reconstruction or repair surgeries.

Exclusion Criteria 

The exclusion criteria for this study consist of several categories of research that are not considered relevant or are deemed less reliable for the purpose of this review. Firstly, animal trials, in vitro trials, cadaveric studies, meta-analysis studies, systematic reviews, and retrospective analyses are excluded. These types of studies are often not directly applicable to human patients and may not provide the same level of insight as studies involving human participants. Secondly, abstracts of randomized controlled trials, case reports, case studies, cohort studies, or technical notes comparing QT, PTBA, and CG in ACL reconstruction or repair surgery are excluded. While these studies may contain valuable information, the abstract format does not provide sufficient detail for a comprehensive review. Thirdly, papers not studying QT, PTBA, or CG in ACL reconstruction or repair surgery are excluded. This ensures that the review focuses specifically on the graft types of interest. Fourthly, papers and abstracts not available in English are excluded from the study. This is due to the challenges of accurately translating and interpreting research findings from other languages. Finally, papers and abstracts published before 2018 are excluded from the study. This ensures that the review focuses on the most recent research and trends in ACL surgery. By excluding older studies, the review aims to provide a more up-to-date and relevant assessment of the current state of ACL reconstruction and repair surgery.

Results

This analysis delves into 20 studies, encompassing randomized controlled trials, prospective cohort studies, and retrospective analyses, to compare the effectiveness and safety of three common graft materials used in ACL reconstruction surgery: allografts, patellar tendon autografts, and CGs. These studies included participant groups ranging from 20 to 1000 individuals.

The upcoming sections of this review will provide a comprehensive summary of the key findings related to the efficacy, outcomes, and complications associated with different graft types used in ACL reconstruction and repair surgery. Firstly, the efficacy section will delve into how effectively each graft type restores stability and function to the repaired ACL. This critical aspect of the review will highlight the performance of each graft material in ensuring the structural integrity and functionality of the knee joint post-surgery. Secondly, the outcomes section will focus on patient recovery progress, return to activity levels, and the long-term joint health observed with each graft type. This section aims to provide insights into the overall recovery trajectory of patients, their ability to resume activities, and the maintenance of joint health over an extended period following ACL reconstruction. Lastly, the complications section will address the potential risks and side effects associated with each graft material. By examining the complications linked to different graft types, this section aims to provide a comprehensive understanding of the safety profile and challenges that may arise post-ACL surgery. By dissecting these aspects, we aim to shed light on the optimal graft choice for ACL reconstruction and repair surgery.

Quadriceps Tendon in ACL Repair Surgery

Table 1 compares different surgical techniques and their outcomes for ACL reconstruction. It illustrates the nature of the study, such as comparing pain and clinical outcomes between patients who received different types of grafts. Additionally, it lists the aim of the study, which is to evaluate the isokinetic peak torque, average power, and total work during knee extension. Moreover, it shows the sample size for each study, and the essential outcomes, such as reduced peak torque and total work in the operated limb compared to the non-operated limb. Furthermore, it presents the conclusion, such as the overall success of the surgery and the importance of considering potential risks and complications. Table 1 revealed that as essential outcomes, patients treated with QT autograft for ACL repair experienced comparable clinical outcomes and post-operative discomfort to those treated with HT autograft. Furthermore, harvesting a QT autograft for ACL reconstruction requires careful consideration of the anatomy of the QT and patella to avoid the risk of patellar fractures during the procedure. Additionally, novel techniques concerning QT were discovered and can lead to more success of this technique for patients with ACL. Comparison between quadriceps and HT autografts show that both provide similarly positive outcomes.

**Table 1:** Recently published studies about quadriceps tendon (QT) allograft from 2018 to 2024.

Type of the study	Aim of the study	Sample size and patient characteristics	Essential outcomes	Conclusion	Year of the study and citation
A randomized controlled trial	Comparing the pain and clinical outcomes between patients with anterior cruciate ligament (ACL) injury treated with quadriceps tendon (QT) allograft and hamstring tendon (HT) autograft, the following can be considered:	28 patients with a primary ACL injury	Between the HT and QT groups, there were no discernible changes in VAS pain, Lysholm knee and Tegner activity scale scores, or IKDC score at any point in time. Positive results were obtained for every patient, and their evaluation ratings increased dramatically.	Patients treated with QT autograft for ACL repair experienced comparable clinical outcomes and post-operative discomfort to those treated with HT autograft.	2020 [16]
Cohort study	In patients receiving FT-Q or PT-Q grafts for ACLR, evaluate isokinetic peak torque, average power, and total work during knee extension.	26 patients who underwent ACL with either an partial-thickness quadriceps tendon (PT-Q) and full-thickness quadriceps tendon (FT-Q) graft were recruited between June 2021 and November 2021	The operated limb exhibited significantly lower peak torque, average power, and total work compared to the non-operated limb in the FT-Q group, but no such differences were observed in the PT-Q group. Specifically, for the FT-Q group, the operated limb showed reduced peak torque, average power, and total work, while the PT-Q group did not display any significant differences in these measures between the operated and non-operated limbs.	The isokinetic variables for the operated limb were significantly lower than the non-operated limb in the FT-Q group. However, there were no significant differences between the non-operated and operated limbs in the PT-Q group.	2023 [17]
Case series; Level of evidence, 4.	Examine the surgical techniques and considerations essential for the secure harvesting of a quadriceps tendon autograft during ACL reconstruction, particularly emphasizing the potential risks of patellar fractures associated with this procedure.	57 patients underwent ACL reconstruction with a quadriceps tendon autograft with a patellar bone block	The incidence of patellar fractures was 3.5% during surgery and 8.8% at the 2-year follow-up. This included 2 fractures that occurred during surgery, 1 fracture that occurred during strength testing, and 2 occult fractures that were detected on computed tomography (CT) scans performed 6 months after surgery for research purposes in asymptomatic participants. Of the 5 patients with patellar fractures who had 24-month follow-up data, the IKDC scores were 91.95, 91.95, 100.00, 100.00, and 64.37.	Harvesting a quadriceps tendon autograft for ACL reconstruction requires careful consideration of the anatomy of the quadriceps tendon and patella to avoid the risk of patellar fractures during the procedure. Postoperative CT imaging may reveal abnormalities in patients who are otherwise asymptomatic, highlighting the importance of considering the potential consequences of this procedure.	2019 [18]
Technical note	Introducing a novel, minimally invasive technique for extracting a QT graft and patellar bone block, preserving the deep QT layer.	One patient	This technique reduces surgical complications during the procedure and leads to better results after surgery.	This technique reduces surgical complications during the procedure and leads to better results after surgery.	2021 [19]
Case report	Provide a supplementary technical comment that emphasizes the use of direct vision with endoscopy for safe, minimally invasive QT harvesting.	One patient under regional or general anesthesia, the patient is placed in the supine position on the operating table and examined.	The surge in popularity of this graft has spurred the creation of less invasive harvesting methods. However, harvesting QT grafts remains a complex procedure with a significant learning curve.	This technical note outlines the endoscopic approach to QT graft harvesting, with the goal of assisting surgeons in safely harvesting the graft while minimizing complications.	2023 [20]
Randomized controlled trial	Conducted a randomized controlled trial (RCT) comparing the outcomes of ACLR using either quadriceps QT or semitendinosus/gracilis hamstring (STG) graft.	One patient	At 2-year follow-up, there were no significant differences between the QT and STG graft groups in terms of subjective patient outcomes, knee stability, or reoperation rates. Donor Site Morbidity At 2 years, donor site symptoms were reported by 27% of patients in the QT group and 50% of patients in the STG group. The donor site morbidity score was 14 for the QT group and 22 for the STG group. Hop Test The hop test demonstrated lower limb symmetry of 91% for the QT graft group and 97% for the STG graft group.	The use of the quadriceps tendon–patellar autograft has shown impressive stability and positive outcomes reported by patients, making it a dependable option for initial ACL reconstruction in adolescent patients.	2019 [21]
Cohort study; Level of evidence, 3.	To compare the results of patients who underwent revision anterior cruciate ligament reconstruction (ACLR) using the double-layer quadruple semitendinosus and gracilis (dlQUAD) tendons technique with those who underwent revision ACLR using a hamstring tendon graft (HT).	A total of 114 patients who underwent revision ACLR	The study identified nine patients who experienced a failed revision ACLR. The group that received the dlQUAD had a notably lower failure rate compared to the HT group. Furthermore, the mean postoperative SSD was significantly lower in the dlQUAD group. At the latest follow-up, the dlQUAD group had significantly better Tegner and IKDC scores than the HT group, indicating an improvement in their condition. Additionally, the dlQUAD group had a significantly lower pain VAS score than the HT group.	Both the dlQUAD and HT techniques showed significant improvement in knee laxity before surgery, and the patient-reported outcome measures after the revision ACLR were satisfactory.	2021 [22]
A case report	The objective of this series of cases study was to assess the outcomes of autologous tendon augmentation in combination with quadriceps tendon repair in patients with chronic kidney disease.	A group of six patients with chronic kidney disease.	At the final follow-up, the average Lysholm score was 93 ± 6.09, with a range of 85 to 100. There were no instances of extension lag observed in this study, and the average range of knee flexion was 134.16 ± 7.63, with a range of 125 to 145.	The use of autologous tendon graft as an augmentation for quadriceps tendon repair showed favorable outcomes ranging from good to excellent, and with no observed cases of re-rupture within a short-term follow-up period. This evidence is at the therapeutic level IV.	2023 [23]
A case report	The following report provides a comprehensive description of the rehabilitation exercise program and post-operative therapeutic objectives and recommendations for patients who undergo surgical treatment for quadriceps tendon rupture, aimed at facilitating their return to daily activities and participation in sports.	A single case report. During a trek through muddy terrain, an active 53-year-old male abruptly stumbled into a hole with his left foot, resulting in the injury.	To ensure rapid recovery and good functional outcomes, a carefully planned rehabilitation exercise program was executed. The patient's postoperative course progressed as expected, with a return to normal daily activities within 6 weeks, achieving full active range of motion by 16 weeks, and resuming sports and recreational activities at 5 months.	Early surgical intervention and a comprehensive rehabilitation program facilitated the patient's recovery, enabling them to: Regain optimal function Return to daily activities within 7 weeks Participate in non-contact sports after 18 weeks	2019 [24]
A randomized controlled trial	The goal of this randomized controlled trial is to generate data that compare the functional outcome of both types of grafts.	24 to QT reconstruction and 27 patients to QHT reconstruction	86% of patients (44 out of 51) completed the 2-year follow-up. Both hamstring tendon reconstruction and quadriceps tendon reconstruction significantly improved knee stability at all time points, with no difference between the two groups. Manual side-to-side displacement improved by: 4.7 ± 3.0 mm in patients with hamstring tendon reconstruction 5.5 ± 2.9 mm in patients with quadriceps tendon reconstruction	The results of primary ACLR show that both quadriceps and hamstring tendon autografts provide similarly positive outcomes.	2021 [25]

Patellar Tendon Bone Allograft in ACL Repair Surgery

Table 2 summarizes and compares the results of multiple studies on ACL reconstruction utilizing bone-patellar tendon-bone (BPTB) allografts. The rows represent separate research studies/reports that varied in design, from randomized controlled trials to cohort studies to case reports. The columns provide key details about each study: Nature of study describes the type of research design used (e.g., RCT, cohort, case series). Sample size/characteristics outlines the number of patients and any relevant demographic information. Essential outcomes summarize the important clinical results, findings, and metrics evaluated in each study (e.g., functional scores, graft rupture rates). Conclusion states the overall conclusions that can be drawn from the study results. Year and citation provide publication details to identify the original source.

**Table 2:** Recently published studies about patellar tendon bone allograft from 2018 to 2024.

Nature of the study	Case or aim of the study	Sample size and patient characteristics	Essential outcomes	Conclusion	Year of the study and citation
Case control study	This study aims to evaluate the clinical outcomes and stability achieved through ACL reconstruction using a quadriceps tendon-bone patellar tendon-bone (QTPB) allograft. They compared these outcomes with those achieved using a QTPB autograft.	Forty-five patients who received ACL reconstruction with QTPB allograft were individually matched in age, sex, direction of the injured knee and body mass index (BMI) to a control group of 45 patients who received QTPB autograft.	Analysis revealed no significant statistical differences between the QTPB allograft and autograft groups in terms of clinical outcomes, complications, or ligament laxity. Similarly, quadriceps peak extension torque showed no significant differences between the groups, except for a single measurement at 6 months.	The clinical outcome achieved with QTPB allograft was favorable and comparable to QTPB autograft, suggesting that QTPB allograft may serve as a promising alternative for ACL reconstruction in appropriately selected and compliant patients.	2018 [26]
Cohort study	Assess the medium-term clinical outcomes of ACL reconstruction by comparing patients who received bone patellar tendon bone allograft (BPTB) with those treated with hamstring autograft (GST).	A total of 43 patients were included, with 21 undergoing autograft ACL reconstruction and 22 receiving allografts. Patients underwent individual interviews employing specific evaluation questionnaires, followed by a clinical assessment involving objective functional tests and a thorough knee examination.	Patients who underwent allograft ACL reconstruction resumed normal sporting activities sooner than those treated with autografts. Overall, subjective test data, clinical evaluations, and physical examinations yielded positive results, with no significant differences observed between the two groups. Additionally, 15 patients in the allograft group and 12 in the autograft group agreed to undergo proprioceptive tests, revealing no notable differences between the groups.	During the follow-up evaluation post-ACL reconstruction, both the BPTB allograft and GST autograft patient cohorts demonstrated comparable outcomes in subjective and objective clinical assessments, as well as limb proprioception.	2019 [27]
Case report	Illustrate the favorable mid-term follow-up outcomes of a case involving staged patellar tendon reconstruction. This procedure followed regional flap surgery to address a soft tissue defect on the anterior knee. The reconstruction utilized doubled BPTB allograft in a patient with a history of infected chronic patellar tendon rupture.	A 19-year-old male patient presented to our clinic three weeks following a motorcycle accident.	This case study demonstrates a minimally invasive surgical approach for repairing chronic patellar tendon defects.	This technique utilizes a small incision focused solely on the affected tendon area, minimizing tissue disruption. This method is particularly suitable for patients with soft tissue limitations who may not tolerate extensive surgery.	2021 [28]
Cohort study	This study aims to evaluate patient-reported outcomes in patients aged 50 years and older who underwent ACLR using a bone-patellar tendon-bone (BPTB) allograft, with a minimum 2-year follow-up period.	Series of patients (50) aged 50 and older who underwent ACLR using BPTB allograft by a single surgeon with minimum 2-year follow-up.	The activity levels of these patients significantly improved after surgery. Similarly, other outcome scores demonstrated positive changes. Notably, 72% of patients achieved a satisfactory symptom state on the IKDC scale, and 74% experienced a clinically meaningful improvement in their activity levels based on the Tegner score. Overall, 76% of patients reported good or excellent outcomes, with only 12% reporting fair or poor results.	Patients aged 50 and older who underwent ACLR (anterior cruciate ligament reconstruction) with a BPTB (bone-patellar tendon-bone) allograft tended to report good outcomes and had significantly higher levels of physical activity based on self-reported assessments at least 2 years after surgery.	2021 [29]
Randomized controlled trial; Level of evidence, 2.	The objectives of this study were to evaluate long-term patient-reported outcomes, graft survival, and the risk of osteoarthritis in individuals who underwent ACL reconstruction with and without lateral extra-articular tenodesis (LET).	121 consecutive knees (120 patients) presenting to a single center with an ACL rupture	Approximately two-thirds of patients were still actively participating in pivoting sports. A total of 17 knees (21%) encountered graft failure, with 5 knees (6%) necessitating revision ACLR. There was no notable disparity in graft failure risk between the BTB group (29%) and the BTB-LET group. Notably, lateral tibiofemoral osteoarthritis was significantly more prevalent in the BTB-LET group (59%) compared to the BTB group (22%).	This study found no significant differences in patients' long-term reported outcomes following ACL reconstruction, regardless of whether a lateral extra-articular tenodesis (LET) was used. However, LET may be associated with an increased risk of lateral compartment osteoarthritis in the long term.	2020 [30]
A randomized control study	This study compared the outcomes of two graft options for ACLR: BPTB grafts and four-strand semitendinosus-gracilis grafts.	40 patients (38 men and 02 women) with ACL tearing were selected for the study.	Statistical comparison was conducted between the functional outcomes of both groups to identify any significant advantages of one group over the other.	The results obtained were inconclusive for both groups. Similarly, the return to pre-injury status was comparable in both groups.	2020 [31]
Randomized controlled trial; Level of evidence, 1.	To contrast the 25-year follow-up outcomes following ACL reconstruction utilizing a bone–patellar tendon–bone (BPTB) graft with or without the Kennedy LAD.	100 patients undergoing ACL reconstruction.	No statistically significant differences were observed between the groups in any of these outcomes, nor in the Tegner score, radiological classification of OA, or number of ACL ruptures.	The results of this study found no statistically significant differences between groups for any of the measured outcomes.	2018 [32]
Technical note	The aim of this study was to compare the medium-term clinical outcomes of patients who underwent ACL reconstruction using either a BPTB allograft or hamstring autograft (GST).	43 patients enrolled in the study participated in a personal interview where they completed specific evaluation questionnaires including the Tegner, Lysholm, Knee Injury and Osteoarthritis Outcome Score, and International Knee Documentation Committee forms. They also underwent a clinical evaluation involving objective functional tests such as the Lachman test and pivot-shift test. A physical examination of the knee was additionally performed.	Patients who underwent reconstruction with an allograft returned to normal sporting activity significantly earlier than those with an autograft. Data from subjective questionnaires, clinical exams, and physical exams were overall positive for both groups with no differences observed between them.	Both patient groups who received either a BPTB allograft or GST autograft for ACL reconstruction showed similar outcomes based on subjective patient-reported questionnaires, objective clinical evaluations, and assessments of limb proprioceptive properties at follow-up.	2019 [33]

Some common themes examined across multiple studies include comparing outcomes of BPTB allografts versus autografts or other graft types; clinical evaluations post-ACL reconstruction; return to activity/sport; and short to long-term follow-up periods.

In summary, this table systematically catalogues and organizes evidence from various research studies on ACL reconstruction utilizing BPTB allografts. The goal is to evaluate the safety, efficacy, and patient outcomes associated with this graft choice based on different levels of evidence.

CG in ACL Construction and Repair Surgery

Table 3 shows the success rates of revision ACL repair and reconstruction using bone-patellar tendon-bone (BTB) allografts from a deceased donor. The study compares the outcomes of this type of revision surgery to historical data from patients who underwent primary ACL reconstruction using an autologous BTB graft. The study found that the revision ACL reconstruction had a success rate of 54.7 points on the Lysholm score, a measure of knee function, which improved to an average of 72.3 points at 1 year and 77.4 points at 3 years post-surgery. This technique was deemed to be a safe and viable option, potentially leading to improved patient satisfaction. In comparison, the control group of primary ACL reconstruction using an autologous BTB graft showed a greater improvement, with an average of 64.4 points on the Lysholm score preoperatively, rising to 85.1 points at 1 year and 88.2 points at 3 years post-surgery. The Tegner score, which reflects activity level, also showed a similar pattern of improvement in both groups. The document also includes a separate study that evaluates the feasibility of using a pedicled vascularized bone graft (VBG) rotated from the lumbar spine to the sacrum. Two types of pedicled VBGs were evaluated, including the posterior element VBG, which showed promising results. This study is the first to demonstrate the use of cadaveric evaluations and a case report of this technique.

**Table 3:** Recently published studies about cadaver graft in ACL construction and repair surgery from 2018 to 2024.

Nature of the study	Case or aim of the study	Sample size and patient characteristics	Essential outcomes	Conclusion	Year of the study and citation
Observational Study	To evaluate the success rate of revision ACL reconstruction using a bone-patellar tendon-bone (BTB) allograft derived from a deceased donor. The outcomes achieved in this revision surgery group will be compared to historical data from patients who underwent primary anterior cruciate ligament (ACL) reconstruction using an autologous BTB graft.	34 patients who underwent surgery, with a minimum follow-up of three years.	Revision ACL with Cadaveric BTB Allograft: The Lysholm score, a measure of knee function, improved from an average of 54.7 points pre-surgery to 72.3 points at 1 year and 77.4 points at 3 years post-surgery. The Tegner score, which reflects activity level, declined from 7.7 points (full performance) before injury to 5.8 points after injury, but recovered to 6.5 points by the 3-year follow-up. Primary ACL Reconstruction with Autologous BTB Graft (Control Group): The Lysholm score showed a greater improvement, averaging 64.4 points preoperatively, rising to 85.1 points at 1 year and 88.2 points at 3 years post-surgery. The Tegner score followed a similar pattern to the revision group, with an average pre-injury score of 6.7 points, dropping to 5.1 points post-injury, and reaching 6.2 points at 3 years after surgery.	Revision ACL reconstruction using a cadaveric bone-patellar tendon-bone (BTB) graft appears to be a safe and viable option. While this technique can potentially improve patient satisfaction.	2021 [34]
Case report	This study investigates the feasibility of using a pedicled vascularized bone graft (VBG) rotated from the lumbar spine (L1) to the sacrum (S1) through a posterior surgical approach. VBGs have a proven track record of improving fusion rates in various bone pathologies.	6 cadaveric torsos, two different VBG donor sites were identified and evaluated	Two types of pedicled VBGs were evaluated in this study: Posterior Element VBG (PE-VBG): This graft involved mobilizing the laminae and spinous processes together (en bloc) using a surgical technique called Gill laminectomy. Iliac Crest VBG (IC-VBG): This graft harvested bone from the superior aspect of the hip bone (iliac crest) and relied on the blood supply from another back muscle, the quadratus lumborum.	This study, combining cadaveric evaluations and a case report, is the first to demonstrate the successful application of pedicled vascularized bone grafts (VBGs) in posterior lumbosacral spinal fusions.	2018 [35]
A case report	Conducting a study comparing the tensile strength of autografts used in ACL reconstruction to determine the most suitable autograft for ACL reconstruction based on mechanical properties.	A 58-year-old woman presented with increasing right ankle pain and swelling over a period of a few months.	The case report presented here showcases the successful treatment of a complete rupture of the peroneus brevis tendon through an end-to-end repair technique using an interpositional cadaver tendon graft. This surgical approach is an effective one-stage procedure that allows for the anatomical reconstruction of the peroneal tendon without the need for an additional tendon transfer or tenodesis procedure.	The use of a cadaveric (donated) graft in the reconstruction of the peroneal tendon is a viable surgical option for patients with severe peroneal tendinopathy. This approach can provide anatomical tension, stabilization, and functional restoration of the affected area.	2018 [36]

Patellar Tendon Bone Allograft

PTBA is an alternative option for ACL reconstruction that offers certain advantages over autografts. The primary benefit of using PTBAs is the reduced donor site morbidity, as they do not require harvesting a tendon from the patient's own knee, thereby minimizing potential pain and weakness at the donor site. Additionally, the use of a pre-prepared allograft can potentially shorten the surgical duration compared to harvesting an autograft during the same procedure. Studies have shown that PTBAs can achieve similar outcomes to autografts in terms of knee stability, functional recovery, and return to sports. Regaining a stable knee joint is crucial for successful ACL reconstruction, and patients can experience improvement in daily activities and movement with the use of PTBAs. Athletes may also be able to resume their desired level of athletic participation. However, it's important to note that PTBAs carry a slightly higher risk of complications compared to autografts. While uncommon with modern processing techniques, allografts have a potential for increased risk of complications, including graft failure, graft rejection, and infectious disease transmission. The allograft tissue may not integrate properly with the recipient's bone, leading to graft failure in 4% to 10% of cases. The body's immune system may attack the allograft tissue, with a rejection rate of 1% to 3%. The risk of infectious disease transmission through allografts is extremely low (less than 0.1%), due to rigorous tissue screening and processing protocols.

In summary, PTBAs offer a valuable option for ACL reconstruction, particularly for those who wish to minimize donor site morbidity or shorten surgery time. However, it's essential to be aware of the slightly increased potential for complications compared to autografts, including graft failure, graft rejection, and infectious disease transmission.

Patellar Tendon Allograft

Patellar tendon autografts are known for their exceptional initial stability and high biomechanical strength, making them a popular choice for younger, active individuals who demand high performance from their knee joint. The graft's strong and stable properties make it an attractive option for those seeking optimal stability and function in their repaired ACL. Evidence has demonstrated excellent long-term outcomes with patellar tendon autografts, including improved knee stability, enhanced knee function, and a successful return to sports for athletes. The graft effectively restores stability to the repaired ACL, and patients experience significant improvement in subjective knee function, feeling a greater sense of control and movement in their knee. Athletes often achieve a successful return to their desired level of athletic activity. Nevertheless, it's important to consider the potential for donor site complications with patellar tendon autografts. While a strong graft choice, they can lead to complications at the donor site, including anterior knee pain, patellar tendinitis, and patellar fracture. Some patients experience pain at the front of the knee (anterior knee pain) after surgery, with rates ranging from 10% to 30%. Inflammation of the patellar tendon (patellar tendinitis) can occur in 5% to 15% of cases, and in rare instances (less than 5%), patients may experience a fracture of the kneecap (patella).

Generally, patellar tendon autografts offer a reliable option for ACL reconstruction, particularly for young, active individuals seeking optimal stability and function. However, it's crucial to consider the potential for donor site complications like pain, inflammation, and fracture, which can impact the patient's recovery and overall outcome.

Cadaver Graft

CGs serve as a readily available source of graft material, offering the advantage of potentially reducing wait times for surgery compared to autografts, where harvesting the tendon adds extra surgical time. This accessibility can be beneficial for patients requiring immediate surgery or those seeking to minimize wait times before their ACL reconstruction procedure. Several recent investigations have demonstrated promising results with CGs, showing comparable outcomes to autografts in terms of knee stability and functional recovery. The reconstruction of the ACL effectively restores stability to the knee joint, while patients experience improvements in their ability to perform daily activities and movements post-surgery. In fact, modern tissue processing techniques have significantly reduced the risks associated with CGs, particularly in terms of disease transmission. The risk of infectious disease transmission through CGs is extremely low (less than 0.1%) due to stringent screening and processing protocols. While there is a slightly higher chance (2% to 5% of cases) of the body not fully accepting the CG compared to autografts, advancements in tissue processing have minimized this risk.

Obviously, CGs offer a viable option for ACL reconstruction, particularly in situations where immediate surgery is desired or when minimizing donor site morbidity is a priority. The advancements in tissue processing have significantly alleviated concerns regarding disease transmission. However, it is important to note that there is a slightly higher possibility of the body not fully accepting the CG compared to autografts, which should be considered when weighing the options for ACL reconstruction surgery.

Discussion 

The surgical management of ACL ruptures is recommended to re-establish anterior-posterior and rotatory knee stability, particularly for athletes who wish to resume competitive and pivoting sports [34-37]. The selection of ACL reconstruction (ACLR) involves considering the anatomical, biomechanical, and histological properties of the three most widely used graft options: bone-patellar-tendon-bone (BPTB), hamstring tendon (HT), and QT allografts. The optimal graft should offer an expeditious harvest with low morbidity, rapid graft integration, and mechanical and structural properties similar to the native ACL [38].

The use of QT allografts for ACL repair and reconstruction has gained increasing attention in recent years. Research, as summarized in Table 1, suggests that QT autografts offer several advantages and comparable outcomes to the more traditional HT autografts. Studies comparing QT and HT autografts for ACL reconstruction have demonstrated similar positive results in terms of clinical outcomes and post-operative discomfort [39,40]. Patients treated with either type of graft experience comparable improvements in knee stability, pain levels, and functional scores. This suggests that QT autografts are a viable alternative to HT autografts, offering similar efficacy in restoring knee function after ACL injury. While QT autografts offer promising results, a potential risk associated with their use is patellar fracture during the harvesting procedure [41]. This highlights the importance of careful surgical technique and a thorough understanding of the anatomy of the QT and patella. Surgeons must meticulously plan and execute the harvesting process to minimize the risk of complications and ensure patient safety. In fact, the body of evidence supports the use of QT autografts as a safe and effective option for ACL reconstruction. They offer comparable clinical outcomes and post-operative discomfort to HT autografts while potentially reducing donor site morbidity. Advancements in surgical techniques and minimally invasive approaches further enhance the success and appeal of QT autografts. As research continues, QT autografts are likely to play an increasingly significant role in the management of ACL injuries. The use of QT allograft, which is a tissue transplanted from a donor, has increased significantly in recent years. This is due to the advantages it offers, such as the absence of donor site complications, reduced surgical time, and the ability to obtain a reliable graft size. However, the published research examining the clinical effectiveness of allograft tendons compared to autograft tendons (taken from the patient's own body) has been inconclusive. The purpose of this paper is to provide a comprehensive review of the current clinical evidence available to help guide surgeons in their decision-making process when choosing between allograft and autograft for primary ACL reconstruction. According to a recent clinical review, allograft tendons represent a safe and efficient alternative for revising ACL reconstruction, exhibiting comparable failure rates and no increased risk of infection when compared to autografts, as long as the tissue remains non-irradiated [42].

Bone-patellar tendon-bone (BPTB) is a frequently utilized source for autografts and allografts in ACL reconstruction. While allografts typically have a higher failure rate, concerns such as donor site morbidity and anterior knee pain can arise with BPTB autografts. There is ongoing debate surrounding the functional outcomes, complications, and knee stability associated with these grafts, with previous comparisons often relying on limited sample sizes from case series. Kraeutler et al. (2013) revealed results that indicated statistically significant advantages for autografts in terms of subjective IKDC, Lysholm, Tegner, single-legged hop, and KT-1000 arthrometer. Meanwhile, allografts showed better results in return to preinjury activity level, overall IKDC, pivot shift, and anterior knee pain. However, allograft BPTB had a higher rupture rate compared to autograft (12.7% vs 4.3%), with no significant difference between the two groups for Cincinnati Knee scores. The outcomes of this meta-analysis showed that patients who receive BPTB autografts for ACL reconstruction have lower graft rupture rates, reduced knee laxity, better single-legged hop test results, and overall higher satisfaction levels compared to those who receive allograft BPTB, according to postoperative evaluations [43]. Similarly, Poehling and colleagues have conducted a prospective study comparing the results of primary ACL reconstruction using either Achilles tendon allograft with soft-tissue fixation or the conventional BPTB with interference screw fixation. The final analysis included 41 patients who received soft-tissue allograft reconstruction and 118 patients who underwent autograft bone-patellar tendon-bone reconstruction. Patients were assessed before surgery and at intervals of 1 to 2 weeks, 6 weeks, 3 months, 6 months, and annually for 5 years after the procedure. In the five-year follow-up, the results of ACL reconstruction with allograft and autograft were compared objectively and subjectively. The long-term outcomes were similar for both groups. However, patients who received allografts reported less pain at 1 and 6 weeks post-surgery, better function at 1 week, 3 months, and 1 year, and fewer activity limitations throughout the follow-up period [44]. Smith and his collaborators conducted a retrospective cohort study to investigate the time taken for athletes undergoing ACL reconstruction (ACLR) to reach specific recovery benchmarks, categorized by the type of graft used. The three graft options considered were bone-patellar tendon-bone (BPTB), hamstring tendon (HT) autografts, and soft tissue allografts (ALLO). All participants in the study completed a structured return-to-sport (RTS) program following ACLR. They concluded that athletes who undergo ACL reconstruction using a patellar tendon autograft may take longer to complete rehabilitation, regain quadriceps strength, and meet return-to-sport criteria compared to athletes receiving HT autografts or soft tissue allografts [45].

The analysis in Table 3 illustrates the use of revision ACL reconstruction using CGs, harvested from deceased donors, to repair previously torn ACL. The researchers compared the outcomes of this revision surgery to historical data from patients who underwent primary ACL reconstruction using their own BPTB grafts (autologous grafts). The table also presents a separate study exploring the feasibility of using a pedicled vascularized bone graft (VBG), a bone graft with its own blood supply, for posterior lumbosacral spinal fusion. This minimally explored technique involves rotating the VBG from the lumbar spine to the sacrum through a posterior approach. The study evaluated two types of pedicled VBGs, with the posterior element VBG showing promising results in cadaveric evaluations and a single case report. This research paves the way for further investigation into the potential clinical application of this innovative technique in spinal fusion surgery. Conclusions from these data indicated that revision ACL reconstruction and repair with cadaveric BPTB allografts offers a valuable option for patients needing revision surgery, while pedicled VBGs hold promise as a future approach for spinal fusion. However, both techniques require further research to optimize outcomes and establish their place in standard surgical practice. In this context, Mert et al. (2023) conducted a study comparing the tensile strength of autografts used in ACL reconstruction to determine the most suitable autograft for ACL reconstruction based on mechanical properties. Dissection of cadavers was carried out to extract the Achilles tendons, QTs, hamstring tendons (semitendinosus and gracilis tendons), patellar tendon grafts, and ACLs. To evaluate the tensile strength of each tendon graft, a Shimadzu Autograph AG-IS 100 kN tester was utilized for testing. In this investigation, the smallest mean difference in tensile strength was observed between the ACL and the QT, implying that the use of the QT in ACL reconstruction may result in more favorable outcomes [46].

A recent study (2020) aimed to compare the outcomes of ACLR using different graft types - QT, HT, and patellar tendon (PT)-using data from the Danish Knee Ligament Reconstruction Registry (DKRR). The researchers hypothesized that QT autografts would result in similar objective knee stability and revision rates as HT and PT autografts. The study analyzed data on primary ACLRs performed in Denmark from 2005 to 2017. Outcome measures included Knee injury and Osteoarthritis Outcome Scores (KOOS), Tegner activity scale scores, sagittal knee laxity, pivot-shift tests at 1-year follow-up, and revision rates at 2-year follow-up. The results showed that a total of 531 QT, 14,213 HT, and 1,835 PT ACLR procedures were registered in the DKRR during the study period. Interestingly, the QT autograft was associated with statistically significantly increased knee laxity (1.8 mm) compared to the HT autograft (1.5 mm) and a higher rate of positive pivot-shift tests. Furthermore, the revision rate at 2-year follow-up was significantly higher for QT autografts (4.7%) compared to PT (1.5%) and HT (2.3%) autografts. In conclusion, the study findings suggest that QT autografts for ACLR may not be as favorable as previously thought. Compared to hamstring and patellar tendon autografts, QT autografts were associated with higher revision rates and poorer objective knee stability measures, such as increased laxity and more positive pivot-shift tests. These results call into question the suitability of QT as a graft choice for ACLR and highlight the need for further research to fully understand the long-term outcomes of different graft types [47]. Xerogeanes and his co-authors (2024) indicated that in the past 12 years, the identification of specific risk factors such as age, activity level, and laxity has prompted research into the failure of ACLR, particularly comparing patellar tendon (PT) and hamstring (HS) tendon grafts. Most studies have shown the PT graft to be superior in outcomes. Concurrently, there has been growing interest in the use of QT autografts for ACL reconstruction, leading to direct comparisons with HS and QT autografts. Once again, the HS tendon has shown inferior outcomes compared to the QT in these studies. Higher laxity measurements appear to be associated with reduced evidence of radiographic healing on magnetic resonance imaging, potentially signaling a shift away from HS graft use in young, at-risk athletic populations [48]. According to recent literature, using the QT autograft for ACL gives comparable clinical outcomes to other autografts while causing less harm at the donor site. Nevertheless, more research is required with longer follow-up and higher levels of evidence to recognize particular groups that may benefit more from the QT [49].

Generally, the choice of graft in ACL reconstruction should be individualized to the patient's anatomy, sport, level of competition, age, risk factors for failure, and graft used in previous ACL surgery. Autografts remain the preferred graft choice in the young athletic patient population due to higher rates of failure, increased costs, and risk of repeat rupture associated with ACL allograft reconstruction [50]. The BPTB autograft consists of a portion of the central aspect of the patellar tendon with its corresponding bone plugs, while the QT autograft is harvested from the QT with or without a bone block. The choice of graft should be tailored to the patient's specific needs and circumstances, with careful consideration given to the potential risks and benefits of each option.

Limitations of the study

This review has several limitations that warrant acknowledgment. Firstly, the inclusion of diverse study designs, such as randomized controlled trials (RCTs), cohort studies, case reports, and technical notes, introduces heterogeneity, making direct comparisons and drawing definitive conclusions challenging [51]. Furthermore, the study only considered three types of grafts and did not explore other potential options, such as synthetic grafts, which could be relevant for a more comprehensive analysis of ACL repair and reconstruction surgery. Additionally, the search strategy focused on studies published within the last six years, potentially excluding relevant older research and limiting the scope of historical data [52]. Moreover, the review primarily focused on clinical outcomes and complications, with less emphasis on cost-effectiveness, patient-reported satisfaction, and long-term functional outcomes like the development of osteoarthritis. The analysis did not account for potential variations in surgical techniques and rehabilitation protocols, which could influence outcomes and introduce confounding factors [53].

Recently, a meta-analysis by Caiano L et al. (2021) compared the clinical outcomes of 785 patients from eight RCTs undergoing primary ACLR with HT autografts versus soft-tissue allografts. The study found a significant difference in favor of HT autografts in patient-reported functional outcomes, but no difference in knee stability and function between the two groups [54]. However, the main limitations of this study were that the soft tissue allograft donor sites were not standardized, patients had different rehabilitation protocols, and follow-up times varied markedly between the studies included. In addition, the review did not address the use of synthetic grafts for ACLR, which have been developed to undertake direct ACLRs and indirect reinforcements of HT or bone-patellar tendon-bone (BPTB) autografts for ACLR [55]. While synthetic grafts provide tensile strength with a maximum load to failure force that exceeds that of the native ACL, there is limited evidence on the strength of the synthetic grafts within the knee joint, and synthetic debris may affect the function of the knee joint, promote synovitis, and increase the risk of revision surgery. Lastly, the review did not discuss the influence of graft choice on the rate of postoperative surgical site infections (SSIs). A recent large, single-center study showed that HT and allograft are associated with a five times higher risk of SSI compared to BPTB [56]. In conclusion, further research with standardized methodologies and longer follow-up periods is needed to address these limitations and provide more definitive evidence on the optimal graft choice for ACL reconstruction.

## Conclusions

In conclusion, this analysis examines three graft options for ACL reconstruction surgery: allografts, patellar tendon autografts, and cadaver grafts. It explores 20 studies to compare their effectiveness, recovery outcomes, and potential complications. Patellar tendon allografts offer advantages like less pain at the graft harvest site and potentially shorter surgery times. Studies show similar success rates in terms of knee stability, function, and returning to sports compared to autografts. However, allografts carry a slightly higher risk of complications like graft failure, rejection, or disease transmission (all very low risks with modern processing). This systematic review details the findings for each graft type to help determine the best choice for individual patients.

Briefly, the selection of graft material for ACL reconstruction surgery should be a well-informed decision, considering patient-specific factors, the expertise of the surgeon, and the existing body of evidence. While allografts, patellar tendon autografts, and cadaver grafts offer distinct advantages and drawbacks, there is no universal solution. Patient age, activity level, underlying health conditions, and the desired surgical outcomes should all play a pivotal role in determining the most suitable graft type to enhance results and reduce risks. It is imperative that ongoing research and advancements in graft materials and surgical methodologies continue to drive progress in ACL reconstruction and repair. By staying at the forefront of innovation, the field can strive towards refining techniques, enhancing patient outcomes, and minimizing complications associated with ACL surgery. This dedication to improvement underscores the importance of a tailored, patient-centered approach in graft selection, ensuring that each individual receives the most appropriate treatment for their unique circumstances.
